# Clinical significance of clusterin expression in pancreatic adenocarcinoma

**DOI:** 10.1186/1477-7819-10-146

**Published:** 2012-07-16

**Authors:** Junshuo Jin, Joon-Mee Kim, Yoon-Seok Hur, Won Pyo Cho, Keon-Young Lee, Seung-Ik Ahn, Kee Chun Hong, In-Sun Park

**Affiliations:** 1 Department of Hepatobiliary Surgery, The First Affiliated Hospital of Zhengzhou University, No. 100 of Science Road, Zhengzhou, Henan, 450001, China; 2 Department of Pathology, Inha University School of Medicine, 7-206, 3-Ga Sinheung-Dong, Jung-Gu, Incheon, 400-711, Republic of Korea; 3 Department of Surgery, Inha University School of Medicine, 7-206, 3-Ga Sinheung-Dong, Jung-Gu, Incheon, 400-711, Republic of Korea; 4 Department of Anatomy, Inha University School of Medicine, 7-206, 3-Ga Sinheung-Dong, Jung-Gu, Incheon, 400-711, Republic of Korea

**Keywords:** Pancreas, Adenocarcinoma, Clusterin, Survival

## Abstract

**Background:**

Clusterin is known to be expressed in many human neoplasms, and is believed to participate in the regeneration, migration, and anti-apoptosis of tumor cells. However, few reports have addressed the relationship between the manifestation of clusterin and clinicopathologic parameters in pancreas cancer patients. In the present study, the authors investigated the expression of clusterin and its clinical significance in pancreatic adenocarcinoma.

**Methods:**

Immunohistochemical staining was performed for clusterin in tumor tissues obtained from patients who received pancreatic resection with radical intent, and the associations of clusterin expression with various clinicopathologic parameters were analyzed in addition to the relation between its expression and survival.

**Results:**

Immunoreactivity for clusterin was observed in 17 of the 52 (33%) pancreatic adenocarcinomas examined. In addition, clusterin positivity was found to be associated with preoperative serum carcinoembryonic antigen level, perineural invasion, and, most strongly, lymph node metastasis. The survival analysis identified tumor differentiation and lymph node metastasis as the only significant prognostic factors.

**Conclusion:**

Although not an independent prognostic factor, clusterin immunoreactivity can be used in conjunction with lymph node metastasis to predict survival in cases of pancreatic adenocarcinoma.

## Background

Pancreatic cancer is a fatal disease with an annual incidence that approaches its mortality rate [1,2], and the 5-year survival rate is about 5% [3]. Pancreatic adenocarcinoma accounts for most pancreatic cancers, and lymph node metastasis is one of the most significant prognostic factors in pancreatic adenocarcinoma patients. However, the extent of lymph node dissection is highly dependent on the operator and the number of dissected nodes can be small, especially when malignancy is not suspected preoperatively. A new biological predictive marker is therefore needed to supplement lymph node status, which can also be used to evaluate the efficacy of adjuvant treatments.

Clusterin is ubiquitously expressed in al most all mammalian tissues and has been found in all human body fluids analyzed [4]. Furthermore, clusterin is also known to be overexpressed in various cancer tissues, including pancreatic cancer [5]. However, the role played by clusterin in pancreatic cancer cells is still unclear, and its clinical significance has yet to be determined. There are two isoforms of clusterin identified, the secretory and the nuclear isoforms [6]. In the present study, we investigated relations between expression of the secretory isoform of clusterin and clinicopathologic parameters to assess its potential value as a prognostic indicator in pancreatic adenocarcinoma.

## Methods

### Pancreatic cancer samples

Pathologically proven pancreatic adenocarcinoma tissue samples were obtained from 52 consecutive patients who underwent surgical resection with radical intent in Inha University Hospital from July 1997 to June 2008. All samples were collected using protocols approved by the local Institutional Review Board, and informed consents were obtained from all the patients.

### Immunohistochemical analysis

Imunohistochemical staining was performed as described previously [7]. All surgical specimens were fixed in 10% formalin, embedded in paraffin, and consecutively sectioned at 5 μm. Most representative sections from each case were dewaxed in xylene and treated with 0.5% hydrogen peroxide in absolute methanol for 30 minutes to block endogenous peroxidase activity. The sections were then washed in PBS and blocked with normal horse serum for 30 minutes at room temperature. Each tissue section was incubated with goat anti-clusterin antibody (1:800; Santa Cruz Biotechnology, Santa Cruz, CA, USA) overnight at 4°C, washed in PBS, and incubated with biotinylated anti-goat IgG and avidin–biotin complex (Vector Laboratories, Burlingame, CA, USA) as secondary antibody for 1 hour at room temperature. Sections were then developed using diaminobenzidine tetrahydrochloride solution (Sigma-Aldrich Corporation, St Louis, MO, USA) and counterstained with hematoxylin. Islet cells were used as internal positive controls. Any case showing focally or diffusely positive tumor cell cytoplasm was defined as clusterin-positive.

### Clinicopathologic data

Medical records were reviewed retrospectively, and data were collected from a medical database. Survival data were extracted from the Korean MicroData Service System (Statistics Korea, Daejeon, Republic of Korea).

### Statistical analysis

Statistical analysis was performed using SPSS for Windows version 12.0 (SPSS, Chicago, IL, USA). The chi-square test was used to examine the nature of the correlation between clusterin expression and clinicopathologic parameters. Survival rates were determined using the Kaplan–Meier method and survival differences were analyzed using the log-rank test. Cox regression was used to identify prognostic factors by multivariate analysis. *P* <0.05 was considered statistically significant.

## Results

### Demographic data and surgical results

There were 37 male and 15 female patients; a male to female ratio of 2.5:1. The median age was 68 years (range, 44 to 87 years). Tumors were located in the head of the pancreas in 44 cases and in the body and/or tail in eight cases (Table 1). All patients with a tumor in the head of the pancreas were managed by pancreaticoduodenectomy with or without gastric pylorus preservation, and the other eight patients were managed by distal pancreatectomy with splenectomy. One patient with a tumor involving the whole pancreas was managed using a combined procedure; the tumor was classified as a head lesion for the statistical analyses. Five patients succumbed to death within 30 days of surgery; an operative mortality of 9.6%.

**Table 1:** Correlations between clusterin expression and clinicopathologic parameters and univariate analysis of survival in pancreatic adenocarcinoma

**Parameter**	**Number of cases (%)**	**Clusterin expression**		**Survival analysis**	
		**Clusterin-positive cases (%)**	***P*value**	**Median survival (months)**	***P*value**
Overall	52 (100.0)	17 (32.7)		14.9	
Age			0.780		0.029
≥65 years	35 (67.3)	11 (31.4)		10.4	
< 65 years	17 (32.7)	6 (35.3)		38.7	
Sex			0.747		0.792
Male	37 (71.2)	13 (35.1)		15.0	
Female	15 (28.8)	4 (26.7)		10.4	
Tumor size			0.241		0.075
≤2 cm	9 (17.3)	1 (11.1)		35.4	
> 2 cm	43 (82.7)	16 (37.2)		14.7	
Location			0.413		0.648
Head	44 (84.6)	13 (29.5)		15.0	
Body/tail	8 (15.4)	4 (50.0)		9.9	
Pathologic grade			0.711		0.010
Well/moderately differentiated	42 (80.8)	13 (31.0)		15.9	
Poorly differentiated/anaplastic	10 (19.2)	4 (40.0)		3.1	
Perineural invasion			0.042		0.414
No	8 (15.4)	0 (0.0)		18.5	
Yes	44 (84.6)	17 (32.7)		11.5	
Lymphovascular invasion			0.330		0.055
No	15 (28.8)	3 (20.0)		39.6	
Yes	37 (71.2)	14 (37.8)		8.9	
Preoperative CEA (ng/ml)			0.048		0.787
≤5	32 (69.6)	8 (25.0)		15.0	
> 5	14 (30.4)	8 (57.1)		14.7	
Preoperative CA19-9 (U/ml)			1.000		0.640
≤37	15 (33.3)	5 (33.3)		15.9	
> 37	30 (66.7)	10 (33.3)		16.5	
Lymph node metastasis			0.006		0.002
No	16 (31.4)	1 (6.3)		39.7	
Yes	35 (68.6)	16 (45.7)		9.9	
Clusterin expression			–		0.312
Negative	35 (67.3)	–		15.0	
Positive	17 (32.7)	–		14.9	

CA19-9, carbohydrate antigen 19-9; CEA, carcinoembryonic antigen.

### Expression of clusterin in adenocarcinoma tissues

**Figure 1:**
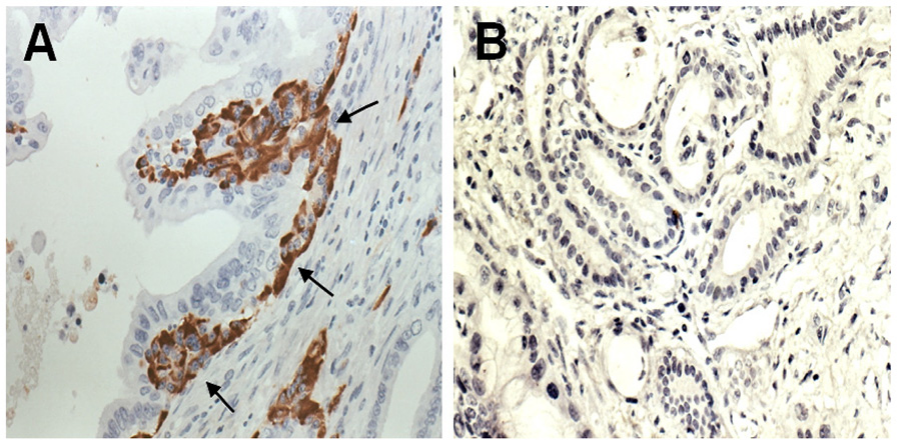
**Clusterin expression in pancreatic adenocarcinoma. (A)** Positive immunohistochemical staining for clusterin in the cytoplasm of ductal cancer cells near the basement membrane (arrows, ×400). **(B)** A clusterin-negative specimen (×200).

Clusterin was expressed in 17 of the 52 (32.7%) pancreatic adenocarcinoma samples. Clusterin was expressed in the cytoplasm of cancer cells in clusterin-positive cancers, (Figure 1a), and was expressed in normal islets but not in adenocarcinoma cells in clusterin-negative cancers (Figure 1b).

### Clusterin expression and clinicopathologic characteristics

Relations between clinicopathologic parameters and clusterin expression were investigated. Clusterin expression was found to be associated significantly with lymph node metastasis (*P* = 0.006), perineural invasion (*P* = 0.042) and elevated preoperative serum carcinoembryonic antigen level (*P* = 0.048), respectively (Table 1).

### Survival analysis

**Figure 2:**
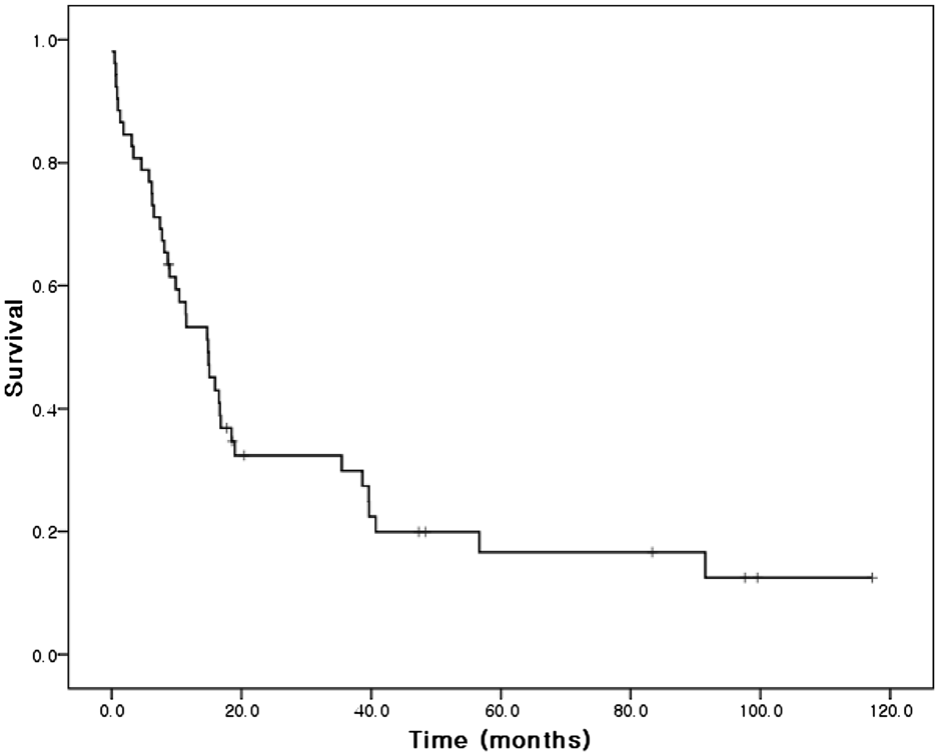
Overall survival curve for the 52 study subjects.

**Figure 3:**
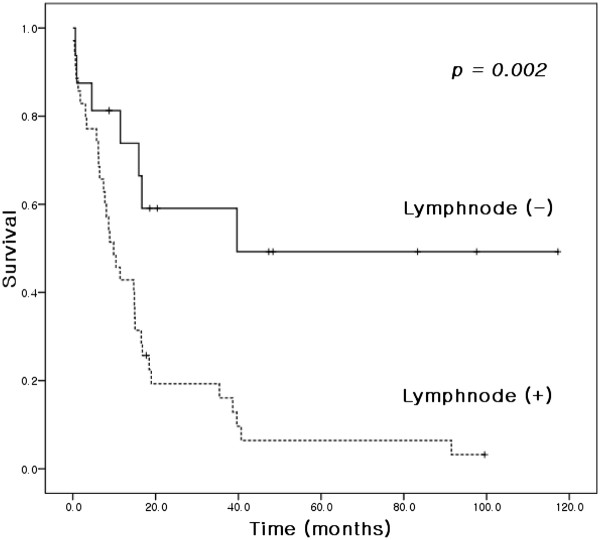
Kaplan–Meier survival curves for patients with or without lymph node metastasis.

**Figure 4:**
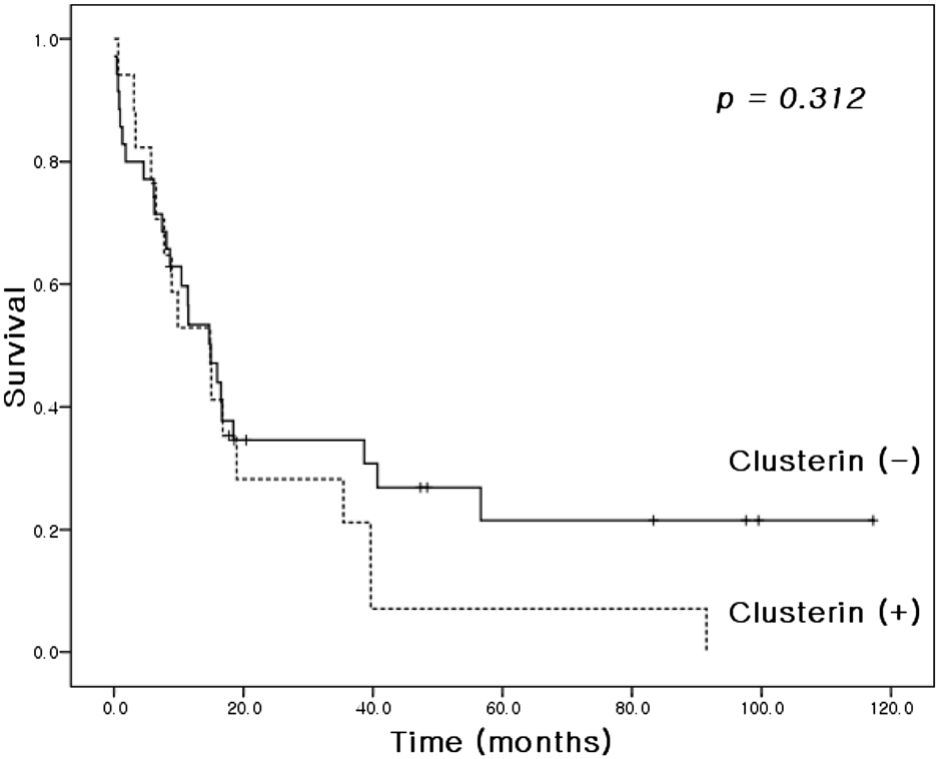
Kaplan–Meier survival curves by clusterin expressional status.

Overall median and 5-year survivals were 14.9 months and 16.6%, respectively (Figure 2). The results of univariate analysis conducted to identify factors associated with survival are shown in Table 1. Age (*P* = 0.029), pathologic grade (*P* = 0.010), and lymph node metastasis (*P* = 0.002) (Figure 3) were found to be significant prognostic factors. Clusterin manifestation was not found to be a significant prognostic indicator (*P* = 0.312; Figure 4), and lymphovascular invasion was only marginally significant (*P* = 0.055). Factors with *P* <0.1 and clusterin status were entered into the Cox regression multivariate analysis (Table 2). This analysis showed that the pathologic grade and lymph node metastasis were significant prognostic factors.

**Table 2:** Multivariate analysis of survival factors

**Factor**	**Hazard ratio**	**95% confidence interval**		***P*value**
		**Lower**	**Upper**	
Age	1.020	0.973	1.068	0.416
Size	1.728	0.559	5.337	0.342
Pathologic grade	3.462	1.251	9.579	0.017
Lymphovascular invasion	1.144	0.490	2.671	0.756
Lymph node metastasis	4.410	1.587	12.256	0.004
Clusterin expression	0.650	0.283	1.494	0.310

## Discussion

Despite recent diagnostic and management advances, pancreatic cancer remains a highly lethal disease [1,2,8,9]. The ability to predict prognosis provides an important means of determining management protocols and follow-up schedules. Of the many clinicopathologic parameters studied, lymph node metastasis has been most consistently associated with prognosis in pancreatic cancer [8,10-12]. Regarding biochemical markers of prognosis, virtually all potential molecules have been tested. Recently, in an extensive review of the literature, Tonini and colleagues concluded that p16, matrix metalloproteinase-7, and vascular endothelial growth factor are probably useful indicators of prognosis [13].

Clusterin is a glycoprotein produced by a wide range of tissues and is present in most body fluids. In its secretory form, clusterin is believed to be involved in many physiologic processes, including apoptosis, morphologic transformation, and cell aggregation [6,14]. The clinical implications of clusterin expression in malignant diseases are controversial, and its contributions at the molecular level remain unclear. Clusterin overexpression has been reported to be marginally related to early recurrence and shorter survival in breast cancer, especially in early stage disease [15,16], and has been shown to increase resistance to anti-estrogen hormonal therapy [17,18]. These results appear to be due to the association between clusterin expression in breast cancer tissues and lymph node metastasis and negativity for hormonal receptors [19,20]. Similarly, clusterin expression in prostate cancer has been correlated with the Gleason tumor grade [21], and is believed to compromise survival by inhibiting apoptosis after hormonal ablation therapy [22]. In a recent study, the possibility that clusterin expression may confer gemcitabine resistance in pancreatic cancer was suggested [23]. In ovarian carcinoma, clusterin overexpression has been shown to be correlated with tumor aggressiveness and/or to be a prognostic factor [24-26]. The results of the present study support the association of clusterin expression with lymph node metastasis and perineural invasion, and thus with aggressive tumor behavior of pancreatic adenocarcinoma. These findings are consistent with those of other studies performed on gastrointestinal adenocarcinomas, in which cytoplasmic clusterin expression was found to be associated with lymph node metastasis and significantly shorter survival in advanced gastric cancer [27], and colorectal cancer [28,29]. The exact mechanism of clusterin overexpression in enhancing cancer metastasis is still unclear. One of the plausible explanations is that clusterin may confer an anti-apoptotic effect on tumor cells once they are detached from the original site, and thus prolongs cell survival under unfavorable conditions in the metastatic process [30].

On the other hand, in an *in vitro* study of nonsmall-cell lung cancer cell lines, clusterin overexpression was found to reduce chemosensitivity but indicated a favorable prognosis due to the inhibition of tumor cell migration [31]. Together with the finding that cytoplasmic clusterin expression is associated with chemoresistance [32], it is presumed that clusterin is basically cytoprotective and favorably affects prognosis in early stage lung cancer but compromises prognosis during late stage disease by conferring resistance to adjuvant radiotherapy and chemotherapy [33]. These seemingly disparate results may be due to a lack of knowledge regarding the causal relationship between clusterin expression and tumor cell apoptosis, which can affect patient survival.

Xie and colleagues recently reported associations between clusterin expression and various pancreatic pathologies including pancreas adenocarcinoma, and concluded that clusterin positivity is related to improved survival, at least by univariate analysis [5]. Their results contradict those of the present study with respect to the direction of the prognostic influence of clusterin expression. This disagreement may have been caused by the small number of cases involved in both studies and a shorter follow-up period in the former study, because in the present study the survival difference between clusterin-positive and clusterin-negative patients became apparent in survivors who had lived longer than 20 months, although the difference could not reach statistical significance.

To date, clusterin has not been found to be an independent prognostic factor for pancreatic adenocarcinoma. However, the results of the present study indicate that clusterin expression is strongly associated with lymph node metastasis, which is one of the most potent prognostic indicators. The impact of clusterin expression on survival can be too weak to be an independent prognostic factor, and further studies with a larger case volume may reveal the statistical significance. While extended lymphadenectomy did not show additional survival benefit [10], regional lymph node dissection still has a role in predicting the patient’s prognosis [11]. There are also cases in which pancreas adenocarcinoma was an incidental finding [34], and the extent of lymph node dissection can be quite varied depending on the operator. Considering these circumstances, clusterin expression may provide an adjunctive means, with regard to the nodal status, in predicting survival, especially when the presence of malignancy is not suspected preoperatively. One of our patients had an occult cancer of the pancreatic head and underwent pancreaticoduodenectomy without lymph node dissection. In this case, the resected specimen was clusterin-negative, and the patient survived 57 months after surgery to succumb to nonsmall-cell lung cancer. The last abdominal computed tomography image in this case showed no evidence of pancreatic cancer recurrence at 47 months after pancreaticoduodenectomy.

## Conclusions

Clusterin expression was not found to be an independent prognostic factor for pancreatic adenocarcinoma, although clusterin-negative patients tended to live longer. A further study on a larger patient population is needed to re-examine the prognostic value of clusterin expression and to identify the underlying mechanisms responsible. Nevertheless, the strong association between clusterin expression and lymph node metastasis suggests that clusterin can be used as an adjunct to lymph node positivity to predict survival in pancreatic cancer.

## Abbreviations

PBS, phosphate-buffered saline.

## Competing interests

The authors declare that they have no competing interests.

## Authors’ contributions

All authors read and approved the final manuscript. JJ carried out the molecular studies. JMK interpreted the pathologic findings. YSH performed the operations. WPC carried out the data collection. KYL performed the operations and drafted the manuscript. SIA performed the operations. KCH performed the operations. ISP directed and interpreted the immunohistochemical stain.
